# A call for a US National Institute of Women’s Health and Human Development

**DOI:** 10.3389/fendo.2024.1289592

**Published:** 2024-03-06

**Authors:** Lawrence M. Nelson

**Affiliations:** Digital Women's Health Initiative, Mary Elizabeth Conover Foundation, Tysons, VA, United States

**Keywords:** women’s health activism, women’s health funding, women’s health policy, women’s health research, digital medical hub, community-based participatory research

## Introduction

Establishing a US National Institute of Women’s Health and Human Development (NIWHD) would serve as a focal point around which to bring women’s health research fully into the digital age. A cloud-based women’s health research hub would provide comprehensive services, including data management, medical records, virtual consultations, and appointment scheduling. It would offer flexibility, convenience, and efficiency to patients and healthcare researchers. One report has demonstrated the feasibility of creating a global digital medical hub for women’s health for one specific rare disorder, Primary Ovarian Insufficiency (POI) (1). A digital medical hub combines the community-building power of social media, peer-reviewed research, and global digital connectivity.

An NIH program is needed to anchor US women’s comprehensive health care with research. An existing US model anchoring care with research is the *NCI Cancer Centers Program*, instituted as part of the National Cancer Act of 1971. The program recognizes centers meeting rigorous standards for transdisciplinary, state-of-the-art care and research (2). A similar *NIH Women’s Health Centers Program* would be ideal to link women’s comprehensive healthcare with research nationwide.

Establishing a US NIWHD would bring several research advantages.

It would:

1. Prioritize research on women’s health issues and generate more funding. These factors would lead to a better understanding of the unique health challenges that women face throughout their lives.
2. Promote gender equity in clinical research by ensuring trials adequately represent women.
3. Facilitate the development of evidence-based interventions to improve women’s health outcomes.
4. Address health disparities women face from marginalized communities by promoting research into the social determinants of health and identifying strategies to manage them.

## Crisis in women’s health research

Albert Einstein said, “You can’t solve a problem on the same level that it was created. You have to rise above it to the next level.” (3) It is time for a fresh perspective on women’s health research. Notably, in, 1993, now 30 years ago, the US National Institutes of Health (NIH) Revitalization Act made it mandatory to include women in research and clinical trials funded by the NIH. Still, today, when viewed from the perspective of disease burden, diseases that predominantly affect women receive a fraction of the funding awarded for conditions that predominantly affect men (4). Structural biases continue to operate (5). The US crisis of maternal mortality rates is a case in point requiring a national research agenda (6). Rising to the next level requires a solid funding base by establishing a US National Institute of Women’s Health and Human Development (NIWHD). The next level described here will focus on unifying care and research in one domain by creating synergy between the communication power of digital health and the integrative capabilities of community-based participatory research (7). Women’s health care and research must be consolidated, coherent, and centered on patient needs.

Unmet needs in women’s health research include conditions that:

- Are specific to women
- Affect women disproportionately
- Affect women differently
- Are inadequately studied or resourced for women

The US healthcare and associated research system is generally dysfunctional, in many ways quietly ruthless, and needs dramatic corrective action (8). The US and the world need stateswomen and statesmen in this arena. A good statesperson is a strong leader, effective communicator, honest, and able to make tough decisions. Improving public health and healthcare requires promoting a general understanding of medical research, supporting policies that advance medical science, generating support for medical research, and implementing public health practices.

## Great expectations

Many in the women’s health community expected the, 1993 US National Institutes of Health (NIH) Revitalization Act to lead to the creation of an NIH institute focused on women’s health. This never happened. The NIH created the Office of Women’s Health Research in, 1990 with a mission “to advance the consideration of women’s health and sex and gender influences across the entire research continuum to improve women’s health.” (9) An office does not have the resources of an institute. An online search to find an explanation of why NIH has never created an institute dedicated to women’s health research did not find even a shred of discussion about this topic. It remains a mystery. It would be refreshing to read an NIH leadership report explaining the reasons behind this decision.

The NIH Office of Research on Women’s Health (ORWH) recognizes that it needs help (10). Their goals are admirable. Yet, an office is not responsible for scientific review, a primary role for an NIH institute, and a critical element in directing funds to the most effective and influential research (11). The NIH ORWH focuses on building a broad-based network of collaborative partnerships with government, nonprofit, academic, and business organizations to integrate sex and gender into biomedical research and achieve optimal health for all women (12). Such an approach is like herding cats. Creating a collaborative space does not consolidate, direct, and manage research funding specific to women’s health.

The next level of women’s health research must focus on unifying care and research in one domain utilizing modern information technology. There have been calls to broaden the concept of women’s health (13). Bridging the divide between clinical research publications and their practical implementation in healthcare is equally important (14, 15).

More than reproductive health, women’s health includes brain, heart, bone health, and beyond. A focus on reproductive health fragments the overall women’s health research agenda. Presently, women’s reproductive health issues undergo scientific review by the National Institute of Child Health and Human Development (NICHD). NICHD is dedicated to advancing research and understanding of human development, reproductive health, and the well-being of children and adolescents, with the vision of promoting healthy pregnancies, healthy children, and healthy and optimal lives (16). Establishing an NIWHD would provide a path to a research agenda that is consolidated, coherent, and centered on the broader aspects of women’s health needs.

## Keep the woman at the center

The “Tragedy of the Commons” occurs when individuals or groups act in their self-interest without regard for the interests of others or the long-term health of the shared resource (17). The concept highlights the importance of resource management and regulation to ensure sustainability and equity (18). Women’s health care and research management must shift to an approach focused on the needs of people and away from dependence on health systems and institutions focused on diseases (19). Women, representing the specific needs of women, must participate actively in the research process and provide collaborative governance. This is community-based participatory research (CBPR) (20), a collaborative research approach involving community members’ active participation in the research process to ensure the research is relevant, culturally appropriate, and leads to meaningful change in the community. CBPR exhibits the following essential elements: community engagement, mutual learning, capacity building, and action-oriented research.

## Stable long-term research funding

The NIH and the NIH Intramural Research Program (NIH-IRP) are able to provide stable funding over several decades to conduct basic and clinical research on specific disorders. Patients bring excellent research questions: “Why did this happen to me?” “What should I do about it?”

The woman-at-the-center approach gains traction with a cross-section of research groups and thus facilitates collaborative interdisciplinary research between NIH institutes. One such example is the NIH-IRP POI research team which completed the only longer-term (3 years) prospective, randomized, double-blinded controlled study on physiologic hormone replacement in POI. In this study, the NIH physiologic hormone replacement protocol (NIH P-HRT) for Overt POI normalized bone density over three years, and women tolerated the hormone replacement well (21). Among many studies made possible, the team published histologic evidence demonstrating follicle luteinization as the primary cause of ovarian follicle dysfunction in these women (22). Also, in collaboration with specialists in positive psychology from Arizona State University, the NIH-IRP team completed a cross-sectional study and a one-year prospective observational study on the psychological vulnerability, emotional health, and coping strategies of women with POI (23, 24). In addition, the team conducted basic physiologic research on human ovarian Graafian follicle function (25) and, by working with a mouse model of autoimmune oophoritis, discovered *Mater*, a maternal effect gene critical to early embryonic development and fertility in female mice (26).

The NIH-IRP POI research team created a microcosm for long-term women’s health research envisioned on a grander scale. The program became well known to referring clinicians and women with the disease. The effort created a steady stream of patients willing to contribute to the research efforts and productive collaborations with investigators in other NIH institutes. The result created a specific paradigm of community-based participatory research supported over the longer term (7). The team collaborated with researchers with expertise in bone, mental health, immunology, endocrinology, genetics, occupational therapy, spiritual care, pharmacology, and basic ovarian physiology. These efforts focused on patient needs, naturally leading to a program of multidisciplinary integrated care and research. In, 2007, the New England Journal of Medicine invited a review of the disorder based on the team’s long-term and integrated research on the condition (27).

## Knowledge management

In addition to stable research funding, the NIH-IRP can provide the longer-term administrative and technical support required to conduct cutting-edge basic and clinical research (28). The NIH-IRP Clinical Research Information System (CRIS) plays a crucial role in research success (29). The system inspires a broader view of the importance of healthcare knowledge management in the digital age and a global perspective (30). One report from the NIH-IRP develops the concept of a Clinical Research Integrated Special Program (CRISP) (31).

A global digital medical hub for women’s healthcare and research would reduce mismanagement and provide efficient information exchange processes among patients, healthcare providers, family caregivers, and investigators. One such report highlights the case of a young African woman who experienced a 10-year delay in the diagnosis of secondary amenorrhea (1). The article also discusses the physiological control systems governing the normal menstrual cycle and the pathophysiology and management of secondary amenorrhea, which can lead to significant morbidity related to estradiol deficiency. A digital medical hub provides authoritative and evidenced-based information to the clinician, and educates the patient, highlighting the need to close the gap between knowledge and action in women’s health.

Secondary amenorrhea can lead to significant health implications for the patient and extended family members. Estradiol deficiency is associated with reduced bone mineral density, cardiovascular disease, depression, and anxiety. Educational health resources have been shown to improve self-management and informed decision-making (32). Mobile health (mHealth) presents a mechanism to close the knowledge-action gap in secondary amenorrhea and other health disorders, increase access to health education, and make care more convenient. The strategy is to focus on the needs and goals of girls and women through community engagement, creating ambassadors who spread positive word of mouth. Many lessons learned from specific prototypes are likely generalizable to the larger women’s health research community.

## Conclusion

Creating a focal point to bring women’s health research fully into the digital age has benefits. Evidence has demonstrated it is feasible to create a global digital medical hub for women’s health, leveraging the power of social media, peer-reviewed research, and global digital connectivity. The goal is to establish an international digital medical home for women, which would serve as a foundation for women’s health research more broadly.

Establishing a US National Institute of Women’s Health and Human Development would provide numerous research benefits. It would prioritize research on women’s health issues and secure more funding, leading to a better understanding of the unique health challenges that women face throughout their lives. Additionally, it would promote gender equity in clinical research by ensuring trials adequately represent women. The institute would also facilitate the development of evidence-based interventions to improve women’s health outcomes. Finally, it would address women’s health disparities in marginalized communities by promoting research into the social determinants of health and identifying strategies to manage them.

## Author contributions

LN: Conceptualization, Writing – original draft, Writing – review & editing.
